# Free and Bound Phenolic Profiles of *Rosa roxburghii* Tratt Leaves and Their Antioxidant and Inhibitory Effects on α-Glucosidase

**DOI:** 10.3389/fnut.2022.922496

**Published:** 2022-06-28

**Authors:** Yuzhe Yang, Wu Li, Wenyan Xian, Wei Huang, Ruili Yang

**Affiliations:** ^1^Guangdong Provincial Key Laboratory of Food Quality and Safety, College of Food Science, South China Agricultural University, Guangzhou, China; ^2^School of Biotechnology and Health Sciences, Wuyi University, Jiangmen, China; ^3^Guangdong Laboratory for Lingnan Modern Agriculture, Guangzhou, China

**Keywords:** phenolic profiles, antioxidant activity, alpha-glucosidase, UPLC-Q-Exactive Orbitrap/MS, *Rosa roxburghii* Tratt leaves

## Abstract

*Rosa roxburghii* Tratt (*R. roxburghii*) tea is a traditional Chinese beverage. This study aims to investigate and compare the phenolics in free and bound forms of two cultivars of *R. roxburghii* leaves, and their bioactivities. The total phenolic content of free and bound fractions was 72.71 and 17.75 mg GAE/g DW in Gui Nong No. 5 (GNN5) and 94.28 and 11.19 mg GAE/g DW in Seedless Cili (SC). A total of 37 phenolic compounds were characterized and quantified by UPLC-Q-Exactive Orbitrap/MS with ellagic acid, quercitrin, isoquercitrin, and quininic acid in free fraction, while gallic acid, ellagic acid, and hyperoside were main compounds in bound fraction. The free fraction with higher phenolic contents also showed excellent performances on antioxidant activities and α-glucosidase inhibitory potency than bound phenolics. Therefore, the results highlight that *R. roxburghii* leaves are a promising source enriched in phenolic constituents for functional beverages and nutritional foods.

## Introduction

*Rosa roxburghii* Tratt (*R. roxburghii*), a member of the *Rosaceae* family, is widely planted in the mountainous and central hilly areas of the southwest and central south regions of China. Currently, Gui Nong No. 5 (GNN5) and Seedless Cili (SC) are the largest two cultivars (1). According to the records of “Encyclopedia of Guizhou” and “A Supplement to the Compendium of Materia Medica,” the fruits, leaves, and roots of *R. roxburghii* are widely used to treat many diseases, such as invigorating spleen and dissolving heat stroke. As a medicinal food homoeologous plant, *R. roxburghii* has recently attracted increasing attention, as it is rich in nutritional and functional constituents. The tea made from *R. roxburghii* leaves not only has a unique tea aroma, but also possesses various health benefits, including antioxidant (2), anti-inflammatory, and lowering blood sugar activities (3). It has been historically used as an edible and medicinal resource for the treatment of diabetes in southwestern China. Based on these advantages, *R. roxburghii* tea has been selected into “the national famous, special and excellent new agricultural products catalog.” These healthcare benefits have been mainly ascribed to high contents of phenolics. Studies on *R. roxburghii* tea have focused on process optimization and functional activities, but there are a few reports on the polyphenol species and contents in them.

As the most abundant secondary metabolites of plants, polyphenols are often widely present in plant and plant-derived products in the daily diet, which have various biological functions, such as antioxidant, anti-inflammatory, and antidiabetic activities (4). In particular, polyphenols strongly inhibit α-glucosidase, which is located on the brush border of the small intestine villi and is a key carbohydrate-hydrolyzing enzyme, mostly the terminal α-bonds of oligosaccharides, form glucose, which is the final stage of carbohydrate digestion (5). Therefore, inhibition of α-glucosidase is an effective method for alleviating postprandial hyperglycemia without diarrhea and other intestinal disturbances caused by current drugs, such as acarbose and miglitol (6). Exploration of effective natural polyphenolic α-glucosidase inhibitors with minimal side effects is required and is considered important for the management of diabetes mellitus.

Polyphenols exist in plants in two main forms, namely, those in the free form are often found in the vacuole of plant cells, and those in the bound form are linked to other natural chemical products through ester linkages, ether linkages, and glycosidic linkages (7, 8). Currently, most studies mainly concentrate on the composition and functional activities of free phenolics than that of bound phenolics. In fact, bound phenolics is the more prevalent form of phenolics in foods. For instance, about 50–95% of phenolic contents were contributed in bound form in vegetables (9), fruits, and legume/seeds (10). The significant amounts of bound phenolics released slowly and continuously by the digestion and colonic fermentation may allow improving the bioaccessibility, bioavailability, and bioactivity of phenolics for a comparatively long time. As a result, both free and bound phenolics should be considered to fully estimate the bioactive values of foods, which is conducive to the rational development and utilization of natural chemical resources. However, there are few studies on thorough polyphenols identification based on mass spectrometry and evaluation of antioxidant and α-glucosidase enzyme inhibition activity of *R. roxburghii* leaves. Hence, the characterization of phenolic metabolites of *R. roxburghii* leaves and assessment of their potential biological activities are of great interest in the food and pharmaceutical industries.

Thus, the aim of this study was to characterize the phenolic profile (free and bound) of the largest two cultivars of *R. roxburghii* leaves using ultra-performance liquid chromatography along with Q-Exactive Orbitrap tandem mass spectrometry (UPLC-Q-Exactive Orbitrap/MS). Furthermore, the antioxidant capacities of each phenolic extract were determined by different antioxidant methods and hypoglycemic function was evaluated by the inhibition of α-glucosidase.

## Experiment

### Chemicals

The standard reference materials were purchased from Sigma Aldrich (Shanghai, China). The 2,2’-azinobis [3-ethylbenzothiazoline-6-sulfonic acid ammonium salt (ABTS)], 2,2-diphenyl-1-picrylhydrazyl (DPPH), fluorescein, 6-hydroxy-2,5,7,8-tetramethylchroman-2-carboxylic acid (Trolox), 3’,6’-dihydroxyspiro [isobenzofuran-1 (3 H),9’-(9 H) xanthene]-3-one (FL)2,2’-azobis (2-amidinopropane)dihydrochloride (AAPH), chromato-graphic-grade methanol and Folin-Ciocalteu phenol reagent (2 M), *p*-nitrophenyl-β-D-galactopyranoside (*p*-NPG), α-glucosidase were obtained from Sigma Aldrich (Shanghai, China). Other analytical grade materials were purchased from Tianjin Kemiou Chemical Reagent Co., Ltd. (Tianjin, China).

### Leaf Sample

Leaves of *R. roxburghii* (GNN5 and SC) were harvested from mountainous areas in Guizhou Province (103° 36′ to 109° 35′ E and 24° 37′ to 29° 13′ N) in late December 2020 and precooled and stored at −80°C for 12 h. Using a vacuum lyophilizer (Christ, Osterode am Harz, Germany) and grinder, a fine powder was made and stored at −20°C.

### Extraction of Polyphenols

Free and bound phenolics were extracted according to the method reported by Li et al. (11). For free phenolics, the frozen leaves sample (0.5 g) was mixed with 15 ml of chilled 80% methanol (1% HCl). The mixture was ultrasonicated for 20 min and then centrifuged (4,000 rpm, 20 min, 4°C) to collect the supernatant (three times). The combined supernatants were evaporated using a rotary vacuum evaporator (BUCHI, China) at 35°C, and then dissolved in methanol (final volume, 10 ml) to obtain a free phenolic fraction. After the free phenolic extraction, obtained residues were used to extract bound phenolic compounds by a two-step sequential solvent extraction with base hydrolysis and acid hydrolysis. First, the residue was added to 15 ml NaOH (3 M), after hydrolyzing for 4 h at 30°C under a stream of N_2_, acidified with HCl to pH 2 (6 M), and then centrifuged at 10,000 rpm for 20 min. The supernatant was extracted by equal volumes of ethyl acetate three times. The upper organic phase was collected and evaporated using a rotary evaporator (BUCHI, China). After base hydrolysis, the remaining residue was hydrolyzed with 15 ml of HCl (3 M) for 1 h at 85°C. Once the hydrolysis was completed, this mixture was acidified to pH 2 with NaOH (6 M) and then immediately centrifuged and extracted by the similar procedures described above. The two parts of extracts obtained by alkaline and acid hydrolysis were combined, evaporated, and resolubilized with 10 ml of methanol, which was considered bound phenolics.

### Determination of Total Phenolics Content and Total Flavonoid Content

The total phenolics content was determined by the Folin-Ciocalteu colorimetric method (12). Briefly, 25 μl of sample solution or standard solution and 125 μl of Folin-Ciocalteu reagent were reacted for 10 min at room temperature, and then 125 μl Na_2_CO_3_ was added. The reaction was continued for 30 min after shaking by an oscillator. The absorbance was measured at 765 nm using a spectrophotometer (Molecular Devices, United States). The gallic acid was used as a standard (*y* = 0.0052x + 0.3651, *R*^2^ = 0.9982) and expressed as mg GAE (gallic acid equivalents)/g DW (dry weight) of leaves.

The total flavonoid content of the extracts was determined based on the aluminum chloride colorimetric method as previously described (13). Briefly, 25 μl of sample solution or standard was mixed with 110 μl of NaNO_2_ solution (0.066 M) for 5 min. The mixture was allowed to stand at room temperature for 5 min, and then 15 μl of AlCl_3_ solution (0.75 M) was added and incubated for 6 min. Then, 100 μl of 0.5 M NaOH solution was added. The absorbance was measured immediately at 510 nm by a spectrophotometer (Molecular Devices, United States). The catechin was used as a standard (*y* = 0.002x + 0.0341, *R*^2^ = 0.9973) and expressed as mg CAE (catechin equivalents)/g DW (dry weight) of leaves.

### UPLC–Q–Exactive Orbitrap/MS Analyses

The phenolic compounds of *R. roxburghii* leaf extracts were characterized and quantified using a UPLC system connected to a Q-Exactive Orbitrap MS (Thermofisher Scientific, China) equipped with an electrospray ionization (ESI) source according to the method from our previous study (14). Separation of phenolics was carried out with an ACQUITY UPLC BEH C18 column (2.1 mm × 100 mm, particle size 1.7 μm, Waters, Milford, MA, United States), eluting with solvent A (acetonitrile) and solvent B (0.1% formic acid in Milli-Q grade water) gradient elution as the mobile phase. The gradient was formed as follows: 0–3 min 95–85% B, 3–11 min 85–70% B, 11–15 min 70–50% B, 15–21 min 50–10% B, 21–22 min 10–95% B. The flow rate was 150 μl/min with a sample injection volume of 2 μl, and the column temperature was equilibrated to 20°C. MS experiments work with the following conditions: auxiliary gas (N_2_), 10 arb; sheath gas (N_2_), 35 arb; capillary voltage, 3,200 V; capillary temperature, 320°C; scan range, 100–1,500 m/z.

The total ion current chromatogram was presented in negative ionization mode because of the presence of hydroxyl, glycoside, and/or carboxylic acid groups. Phenolics compounds were identified by comparison of their spectra and retention times with those of externally injected standards such as quininic acid, gallic acid, (-)-gallocatechin, protocatechuic acid, neochlorogenic acid, chlorogenic acid, catechin, cryptochlorogenic acid, *p*-hydroxybenzoic acid, 6,7-dihydroxycoumarin, caffeic acid, benzoic acid, vanillin, rutin, *p*-hydroxy-cinnamic acid, *p*-coumaric acid, ellagic acid, hyperoside, isoquercitrin, ferulic acid, kaempferol -3-*o*-rutinoside, proanthocyanidins, isoferulic acid, astragaline, quercitrin, phlorizin, quercetin, naringenin, kaempferol, and isorhamnetin. For compounds with no available standards, identification was made using the precise mass of the parent ion [(M–H)] and typical MS fragmentation pattern compared with references and OTCML database. The quantification of the characterized phenolic compounds was carried out by the external calibration curve of each standard. When the standard was not available, the compound quantification was expressed as equivalent to the structurally closest phenolic compound.

### Evaluation of Antioxidant Activity

#### Assay of DPPH Radical Scavenging Activity

The DPPH assay was performed by the reported method (15). In dark conditions, a 100 μl sample or blank solution was mixed with 100 μl DPPH (0.35 M) and incubated for 30 min. Its absorbance was measured at 517 nm using a spectrophotometer (Molecular Devices, United States). The result was calculated as mg Trolox equivalents per gram of dry weight (mg TE/g DW) (Trolox standard curve, *y* = 0.0204x − 0.0393, *R*^2^ = 0.9951).

#### Assay of ABTS Radical Scavenging Activity

The ABTS assay was performed as previously described (16). A total of 5 ml 3-ethylbenzothiazoline-6-sulfonic acid diammonium salt (ABTS) (7.0 mM) and 88 μl potassium persulphate (140 mM) were allowed to react for 12 h in dark in order to prepare the fresh stock solution. The ABTS working solution was freshly prepared by diluting 80% methanol to get an absorbance of 0.70 ± 0.02 units at 734 nm. A total of 20 μl sample or blank solution was mixed with 200 μl working solution for 6 min. Its absorbance was measured at 734 nm. The results were calculated as mg Trolox equivalents per gram of dry weight (mg TE/g DW) by comparing with the Trolox standard curve (*y* = 0.0015 x − 0.0492, *R*^2^ = 0.9989).

### Oxygen Radical Absorbance Capacity Assay

The oxygen radical absorbance capacity (ORAC) assay was performed by the reported method (17). PBS buffer (pH 7.4) was used as the solvent for reagents. A total of 100 μl fluorescein sodium solution (8 mM) and 25 μl sample solution or blank solution were mixed well in a black 96-well plate, and the plate was equilibrated at 37°C for 15 min; then, 75 μl AAPH (119.4 mM) solution was added and recording of fluorescence intensity was started by scheduled recording function of a fluorometer with excitation at 485 nm and emission at 530 nm every 2 min for 2 h. The results were expressed as μmol Trolox equivalents per gram of dry weight (μmol TE/g DW) (Trolox standard curve, *y* = 303934 x − 647020, *R*^2^ = 0.9966).

### α-Glucosidase Activity *in vitro*

The α-glucosidase inhibitory activity *in vitro* was estimated by the method previously described (18). At 37°C, 20 μl different concentrations of sample or control solution were incubated with 45 μl α-glucosidase (1 U/ml) in 0.1 M pH = 6.8 phosphate buffer solution for 10 min, and the reaction was continued for 20 min after 45 μl *p*-NPG (2.5 mM) in 0.1 M. Phosphate buffer solution with pH = 6.8 was added. After the addition of 100 μl sodium carbonate (0.2 M), its absorbance was measured at 405 nm. In the control group, PBS buffer was used instead of the sample. Acarbose (0.625–20 mg/ml) was used as the positive control. The α-glucosidase inhibiting activity of the extracts was repressed as a half-inhibition concentration (IC_50_) value. The α-glucosidase inhibiting activity was calculated according to the following formula:


α-Glucosidaseinhibitoryactivity(%)=[1-(A/s⁢a⁢m⁢p⁢l⁢eA)c⁢o⁢n⁢t⁢r⁢o⁢l]×100%.

### Statistical Analysis

All assays were conducted in triplicate and repeated three times, with the data reported as mean ± standard deviation (SD). Data were analyzed by one-way analysis of variance (one-way ANOVA) with Tukey’s HSD test to determine the differences between means. Principal component analysis (PCA) and hierarchical cluster analysis (HCA) were performed and images were generated with Origin 2021b (OriginLab, Northampton, MA, United States). Correlation analyses between phenolics and bioactivities were performed using standard Pearson correlation. All statistical analyses were carried out at a significance level of 5% (*p* ≤ 0.05) using IBM SPSS.

## Results and Discussion

### Total Phenolic Content and Total Flavonoid Content

**FIGURE 1:**
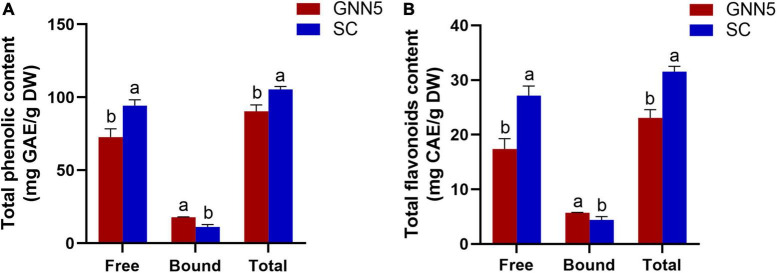
Total phenolic contents **(A)** and total flavonoid contents **(B)** of free and bound phenolic fractions extracted from two *R. roxburghii* leaves. Data represent the mean values ± SD (*n* = 3), and different letters indicate significant difference (*p* < 0.05) between two cultivars. Total, the sum of free and bound fractions.

*Rosa roxburghii* leaves rich in phenolics are traditionally processed into healthy beverages, such as tea. However, the qualitative and quantitative analysis of polyphenols in *R. roxburghii* leaves is limited. Figure 1 shows the phenolic content (free, bound, and total phenolics, and the total phenolic content is the sum of free and bound fractions) of two cultivars of *R. roxburghii* leaves. The free phenolic content of SC was 94.28 ± 4.01 mg GAE/g DW, which was significantly higher than that of GNN5 with 72.71 ± 5.67 mg GAE/g DW, while the bound phenolic content of SC (11.19 ± 1.66 mg GAE/g DW) was lower than that of GNN5 (17.75 ± 0.29 mg GAE/g DW). A similar trend was observed for flavonoids, which was 27.14 ± 1.76 and 17.38 ± 1.90 mg CAE/g DW (free fraction) and 4.42 ± 0.06 and 5.71 ± 0.06 mg CAE/g DW (bound fraction) for SC and GNN5, respectively (Figure 1).

The bound phenolics contributed to 10.61% and 19.62% of TPC in SC and GNN5, resepctively, indicating that there also existed a considerable amount of bound phenolics in addition to the free phenolics in *R. roxburghii* leaves. Importantly, the average sum of TPC (free + bound) of these two cultivars was 97.97 mg GAE/g DW, which suggested that *R. roxburghii* leaves are indeed a valuable source of phenolic compounds compared with other foods. For example, these values are greater than that of 14.16–14.54 mg GAE/g DW in mint (19) and comparable to that of 34.78–40.33 and 87.59–88.27 mg GAE/g DW in green tea (20) and raw black tea leaves (21), which were the well-known natural good source of phenolic compounds.

### Characterization of Phenolic Compounds

Considering the presence of some possible interfering compounds that react with Folin-Ciocalteu reagent, such as sugars, aromatic amines, and organic acids (22), UPLC-Q-Exactive Orbitrap/MS analysis was conducted to further identify and quantify the compositions of phenolic compounds. A total of 37 individual phenolics were identified in all samples of *R. roxburghii* leaves (Supplementary Table 1 and Supplementary Figure 1). According to the reference standards (retention times, the accurate mass, and the secondary fragment), 30 individual phenolics were directly identified. They included quininic acid, gallic acid, (-)-gallocatechin, protocatechuic acid, neochlorogenic acid, chlorogenic acid, catechin, cryptochlorogenic acid, *p*-hydroxybenzoic acid, 6,7-dihydroxycoumarin, caffeic acid, benzoic acid, vanillin, rutin, *p*-hydroxy-cinnamic acid, *p*-coumaric acid, ellagic acid, hyperoside, isoquercitrin, ferulic acid, kaempferol -3-*o*-rutinoside, proanthocyanidins, isoferulic acid, astragaline, quercitrin, phlorizin, quercetin, naringenin, kaempferol, and isorhamnetin. The other seven peaks were tentatively identified by comparing their MS data with those reported in the literature. Peak 7 was characterized as brevifolin carboxylic acid by an [M-H]^–^ ion at m/z 291.0148 and fragment ions at m/z 247.0246 due to the loss of carboxyl moieties (23). Peak 11 (m/z 951.0756) was identified as geranium from its fragment ions at m/z 933.0653 and m/z 300.9991 (24), based on the similar fragmentation pattern reported previously. Peak 12 was preliminarily authenticated as corilagin with parent ion m/z 633.0742 (M–H)^–^ and the fragment ions at m/z 300.9991 (C_14_H_5_O_8_^–^) and m/z 275.0198 (C_13_H_7_O_7_^–^) associated with hexahydroxydiphenoyl group of corilagin (25). Peak 16 (m/z 933.065) may be associated with castalagin, with the ions at m/z 915.0599 indicating fragmentation of these core molecules after the release of one water molecule. Peak 26 (m/z 505.0993) was tentatively characterized as quercetin 3-*O*-6″-acetylglucoside because of its fragment ions at m/z 301.0348, corresponding to the quercetin moiety (26). Peak 29 and peak 32 were identified as kaemperol-3-*O*-glucuronide and kaempferol-pentoside, respectively, at their [M-H]^–^ ion and the fragment ions at m/z 285.0389 (27). Among the abundant individual phenolics, protocatechuic acid, neochlorogenic acid, brevifolin carboxylic acid, catechin, benzoic acid, ellagic acid, hyperoside, ferulic acid, isoferulic acid, astragaline, quercitrin, quercetin, naringenin, and kaempferol derivative were detected in the free and bound phenolic extracts. (-)-Gallocatechin, chlorogenic acid, corilagin, 6,7-dihydroxycoumarin, castalagin, rutin, quercetin 3-*O*-6″-acetylglucoside, and phlorizin derivative were found only in the extracts of free phenolics. In addition, caffeic acid, *p*-hydroxy-cinnamic acid, and *p*-coumaric acid derivatives were found only in the extracts of bound phenolics (Table 1). Interestingly, many individual phenolics detected in *R. roxburghii* leaves such as gallic acid, catechin, rutin, *p*-coumaric acid, hyperoside, isoquercitrin, and kaempferol also abundantly exist in green tea (20).

**TABLE 1:** Phenolic profiles of two *R. roxburghii* leaves in free and bound fractions (mg/100 g DW).

Phenolic compound	GNN5		SC	
		
	Free	Bound	Free	Bound
Quininic acid	138.60 ± 2.56^a^	4.25 ± 0.28^c^	110.42 ± 2.46^b^	5.31 ± 1.33^c^
Gallic acid	3.29 ± 0.22^c^	281.59 ± 2.95^a^	7.27 ± 0.37^c^	132.28 ± 5.91^b^
Protocatechuic acid	0.46 ± 0.06^c^	3.39 ± 0.22^a^	0.20 ± 0.05^c^	2.50 ± 0.29^b^
Neochlorogenic acid	16.10 ± 0.68^b^	1.24 ± 0.22^c^	64.16 ± 1.67^a^	0.31 ± 0.09^c^
Chlorogenic acid	1.08 ± 0.08^b^	ND	25.25 ± 0.40^a^	ND
Brevifolin carboxylic acid	71.85 ± 8.32^b^	11.76 ± 0.80^c^	100.68 ± 3.43^a^	9.59 ± 0.36^c^
Cryptochlorogenic acid	1.39 ± 0.08^a^	ND	ND	0.06 ± 0.00^b^
*p*-Hydroxybenzoic acid	ND	63.25 ± 2.35^a^	ND	ND
Caffeic acid	ND	11.91 ± 0.99^b^	ND	15.09 ± 0.62^a^
Benzoic acid	8.65 ± 0.17^c^	34.27 ± 1.73^a^	6.41 ± 0.15^c^	29.22 ± 3.21^b^
*p*-Hydroxy-cinnamic acid	ND	6.72 ± 0.18^a^	ND	7.54 ± 0.64^a^
*p*-Coumaric acid	ND	7.17 ± 0.21^b^	ND	7.71 ± 0.37^a^
Ellagic acid	237.62 ± 3.95^b^	182.60 ± 5.10^c^	264.21 ± 6.13^a^	179.72 ± 5.33^c^
Ferulic acid	0.88 ± 0.05^c^	11.00 ± 0.11^a^	0.07 ± 0.02*^d^*	3.58 ± 0.26^b^
Isoferulic acid	1.97 ± 0.09^c^	26.99 ± 1.49^a^	0.44 ± 0.18*^d^*	13.43 ± 0.56^b^
ΣPhenolic acids	482.02 ± 13.40^c^	644.54 ± 11.92^a^	579.33 ± 9.84^b^	406.2 ± 6.01*^d^*
(-)-Gallocatechin	2.95 ± 0.08^b^	ND	3.95 ± 0.17^a^	ND
Catechin	73.16 ± 1.63^a^	1.60 ± 0.05^c^	66.66 ± 1.19^b^	1.91 ± 0.26^c^
Rutin	0.33 ± 0.04^b^	ND	11.50 ± 0.29^a^	ND
Hyperoside	97.90 ± 2.57^b^	85.64 ± 0.39^c^	176.71 ± 2.10^a^	66.05 ± 5.86*^d^*
Isoquercitrin	125.17 ± 3.30^b^	2.18 ± 0.44^c^	218.34 ± 2.99^a^	ND
Kaempferol -3-*o*-rutinoside	0.17 ± 0.01^c^	ND	6.32 ± 0.07^a^	0.51 ± 0.17^b^
Quercetin 3-*O*-6″-acetylglucoside	2.04 ± 0.14^b^	ND	12.13 ± 0.81^a^	ND
Proanthocyanidins	ND	ND	0.07 ± 0.01^a^	ND
Kaemperol-3-*O*-glucuronide	1.33 ± 0.13^a^	0.22 ± 0.03^b^	ND	ND
Astragaline	10.59 ± 0.41^a^	8.50 ± 0.24^b^	9.57 ± 0.30^b^	4.36 ± 0.16^c^
Quercitrin	153.25 ± 4.08^a^	0.38 ± 0.02*^d^*	138.92 ± 1.98^b^	6.94 ± 0.75^c^
Kaempferol pentoside	0.65 ± 0.06^b^	ND	10.06 ± 0.54^a^	0.42 ± 0.07^b^
Phlorizin	0.33 ± 0.04^b^	ND	0.60 ± 0.06^a^	ND
Quercetin	2.15 ± 0.19^c^	13.61 ± 0.21^a^	2.42 ± 0.15^c^	10.56 ± 0.99^b^
Naringenin	34.04 ± 0.84^a^	13.18 ± 0.54^c^	24.81 ± 0.95^b^	26.11 ± 1.17^b^
Kaempferol	4.47 ± 0.18^c^	28.58 ± 0.73^a^	1.46 ± 0.08*^d^*	15.98 ± 1.05^b^
Isorhamnetin	0.02 ± 0.00*^bc^*	0.21 ± 0.02^a^	ND	0.03 ± 0.00^b^
ΣFlavonoids	506.59 ± 7.68^b^	154.43 ± 0.37^c^	682.00 ± 4.25^a^	134.06 ± 6.48*^d^*
Geranium	ND	ND	0.15 ± 0.04^a^	ND
Corilagin	33.34 ± 1.21^a^	ND	21.18 ± 0.44^b^	ND
6,7-Dihydroxycoumarin	0.06 ± 0.01^b^	ND	0.08 ± 0.02^a^	ND
Castalagin	9.62 ± 0.64^a^	ND	9.52 ± 0.41^a^	ND
Vanillin	0.09 ± 0.01^c^	0.86 ± 0.06^a^	ND	0.51 ± 0.02^b^
ΣOthers	43.11 ± 1.78^a^	0.86 ± 0.06^c^	30.94 ± 0.85^b^	0.51 ± 0.02^c^
ΣPhenolic compounds	1031.72 ± 22.01^b^	799.83 ± 11.61^c^	1292.27 ± 8.75^a^	540.77 ± 8.40*^d^*

*Data represent the mean values ± SD (n = 3). ND, not detected/determined. Brevifolin carboxylic acid, geranium, corilagin, and castalagin were quantified in ellagic acid equivalents; quercetin 3-O-6″-acetylglucoside was quantified in quercetin equivalents; kaemperol-3-O-glucuronide and kaempferol pentoside were quantified in kaempferol equivalents. Different letters in same line indicate significant difference (p < 0.05).*

### Quantitative Analysis of Individual Phenolic Compounds in Various Extracts

The quantitative analysis of individual phenolic compounds from *R. roxburghii* leaves was performed with the corresponding relative standards (Supplementary Table 2). As shown in Table 1, abundant individual phenolics of SC and GNN5 in free form were ellagic acid, quercitrin, quininic acid, isoquercitrin, hyperoside, brevifolin carboxylic acid, and catechin. Compared with those abundant in free phenolics, those abundant phenolics in bound fractions were gallic acid, ellagic acid, and hyperoside for SC and GNN5, respectively. The bound phenolics contributed to 29.50% and 43.67% of total phenolics content in SC and GNN5, respectively. Acosta-Estrada et al. (9) reported that 16.7–76.3% of phenolics compounds exist fundamentally in the bound form in fruits. Considering the effect of bound phenolics on gut health after liberation by microbiota in the colon, exploring bound compounds of *R. roxburghii* leaves is significant to estimating the scientific support for healthcare. In addition, the total concentration of phenolics detected by UPLC-ESI-MS/MS in *R. roxburghii* leaves was up to 1,831.55 mg/100 g DW (GNN5) and 1,833.04 mg/100 g DW (SC), which suggested *R. roxburghii* leaves as a good source of phenolic compounds. The high phenolics (free + bound forms) clearly indicated that *R. roxburghii* leaves have a great potential application in the development of functional and value-added foods. In addition, the abundant individual phenolics of green tea, including catechins, gallic acid, and ellagic acid (28), were also found in *R. roxburghii* leaves.

### Antioxidant Capacity

The healthy function of polyphenols has been mainly ascribed to their antioxidant activity, which can be measured by scavenging or reducing radical species (e.g., DPPH, ABTS, and ORAC assays) (29). Therefore, DPPH, ABTS, and ORAC assays were employed to assess the antioxidant potential of the different phenolic extracts from *R. roxburghii* leaves. As summarized in Table 2, the DPPH values of free phenolics in SC (311.80 ± 3.53 mg TE/g DW) and GNN5 (310.15 ± 4.55 mg TE/g DW) were significantly higher than that of their bound phenolics (28.35 ± 0.52 mg TE/g DW and 50.53 ± 1.43 mg TE/g DW) (*p* < 0.05). However, no significant difference was observed between the two cultivars for free phenolic extracts (*p* > 0.05). With similar results found in ABTS, ABTS values of free phenolics from SC (1,336.87 ± 70.49 mg TE/g DW) and GNN5 (1,355.48 ± 182.13 mg TE/g DW) exhibited 9.03 and 5.41 times that of their bound phenolics, respectively. Moreover, free phenolics of SC exhibited the highest ORAC value (2,017.38 ± 20.95 μmol TE/g DW), followed by free phenolics of GNN5 (1,730.52 ± 8.57 μmol TE/g DW), bound phenolics of GNN5 (839.58 ± 34.95 μmol TE/g DW), and SC (610.25 ± 23.05 μmol TE/g DW). These values are greater than that of 777.00–1,173.00 μmol TE/g in black tea (30) and comparable to that of 934.00–1,302.60 and 1,060.10–1,125.00 μmol TE/g in oolong tea and green tea (31), respectively, which were normally well-known natural antioxidant products. Overproduction of free radicals relates to oxidative stress and plays a significant role in the development of chronic diseases, such as cardiovascular diseases and diabetes, and dietary antioxidants may block the occurrence and progression of these diseases (32). The results indicated that phenolic extracts of *R. roxburghii* leaves may be consumed as natural dietary antioxidants.

**TABLE 2:** Antioxidant capacity of free and bound phenolic compounds extracted from two *R. roxburghii* leaves.

	DPPH (mg TE/g DW)	ABTS (mg TE/g DW)	ORAC (μ mol TE/g DW)
Free phenolics (GNN5)	310.15 ± 4.55^a^	1355.48 ± 182.13^a^	1730.52 ± 8.57^b^
Free phenolics (SC)	311.80 ± 3.53^a^	1336.87 ± 70.49^a^	2017.38 ± 20.95^a^
Bound phenolics (GNN5)	50.53 ± 1.43^b^	250.42 ± 12.74^b^	839.58 ± 34.95^c^
Bound phenolics (SC)	28.35 ± 0.52^c^	147.99 ± 12.16^b^	610.25 ± 23.05^d^

*Data represent the mean values ± SD (n = 3) and different letters in the same column indicate significant difference (p < 0.05).*

### α-Glucosidase Inhibition Activity

**FIGURE 2:**
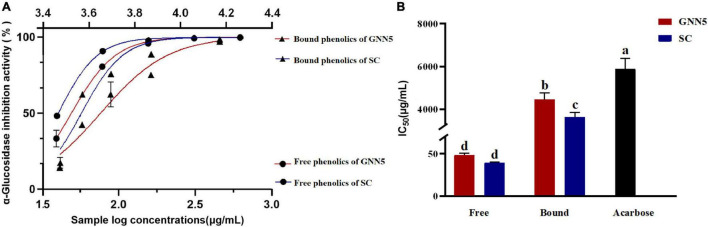
The α-glucosidase inhibitory potency of free and bound phenolic compounds extracted from two *R. roxburghii* leaves **(A)**. Panel **(B)** represents the corresponding IC_50_ values of the samples. Different letters denote statistically significant differences at *p* < 0.05.

Located in the small intestine, the role of α-glucosidase is to hydrolyze glycosidic bonds in oligosaccharides and disaccharides and release D-glucose. Glucosidase inhibitors interfere with the role of α-glucosidase, delay the absorption of D-glucose, and reduce postprandial blood glucose levels (18). So far, the widely used drug product is acarbose; for this experiment, it was used as a positive control. As shown in Figure 2, the polyphenolic extracts of *R. roxburghii* leaves were superior to the positive control in terms of their inhibitory effects. The IC_50_ values of phenolic extracts of *R. roxburghii* leaves were significantly lower than that of the positive control, among which free phenolic extracts of SC (39.72 ± 0.59 μg/ml) exhibited the lowest (*p* < 0.05) IC_50_ value, which was significantly lower by 99.34% compared with the positive control acarbose (IC_50_ = 5.98 mg/ml). The α-glucosidase inhibition activity of free phenolics of GNN5 (IC_50_ = 49.00 ± 1.71 μg/ml) was also significantly lower than that of acarbose, but higher than that of free phenolics of SC. Meanwhile, bound phenolics of SC and GNN5 also possessed higher α-glucosidase inhibitory activity (IC_50_ = 3.711 ± 0.21 and 4.524 ± 0.30 mg/ml), which were also significantly lower than the positive control acarbose. In line with the previous research (33, 34), phenolics from *Rosa roxburghii* leaves also exhibited a strong inhibition against the α-glucosidase activity. Previous studies reported that gallic acid, quercitrin, isoquercitrin, and catechin inhibited the α-glucosidase activity and significantly decreased the hydrolysis rates of different starches (35). Ellagic acid (180 mg/day for 8 weeks), as a strong α-glucosidase inhibitor (36), significantly reduced the blood glucose level and insulin resistance of patients with type 2 diabetes (37). Therefore, the α-glucosidase inhibiting activity of *R. roxburghii* leaf extracts can be mainly attributed to the presence of phenolics compounds. As the raw material of *R. roxburghii* tea, the good inhibitory effect of *R. roxburghii* leaves on glucosidase provided strong evidence for the validation of its functional activity.

### Principal Component Analysis and Hierarchical Cluster Analysis

**FIGURE 3:**
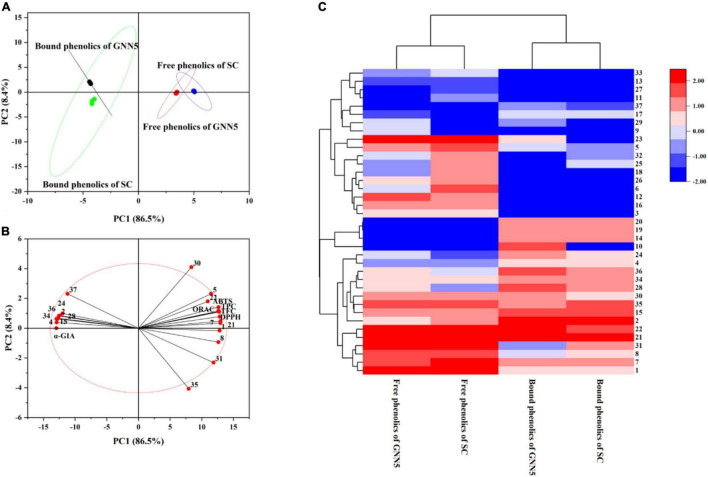
Multivariate analysis on the datasets of two *R. roxburghii* leaf extracts. **(A)** The PCA loading plot; **(B)** the PCA score plot; **(C)** Hierarchically clustered heat (HCA) map. The component numbers are in one-to-one correspondence with Supplementary Table 1.

Principal component analysis, which provided direct visualization of the distribution of individual phenolic compounds and bioactivity of different samples, was performed and the maps were generated (Figures 3A,B). The loading plot of PCA showed that PC1 86.5% and PC2 8.4% accounted for 94.9% of the total variances, which indicated that these two principal components could load maximum information from the original data. The free phenolics obtained from GNN5 and SC were classified into cluster 1, while the bound phenolics extracted from these two cultivars were classified into cluster 2. Two clusters from the same phenolic fraction of different cultivars with intersections indicated that their compositions were similar. With respect to the score plot of PCA, the cosine values may indicate the relationship between two variables (Figure 3B). The results confirmed that the bioactivities of phenolic extracts from *R. roxburghii* leaves were evidently influenced by TPC and TFC. Among them, quininic acid (1), neochlorogenic acid (5), brevifolin carboxylic acid (7), ellagic acid (21), and hyperoside (22) were significantly correlated with ABTS and DPPH scavenging capacity and ORAC (the antioxidant activities). In addition, quininic acid (1), brevifolin carboxylic acid (7), and catechin (8) were significantly correlated with the α-glucosidase inhibiting activity. This study highlighted the significant effect of phenolics on the biological activities of *R. roxburghii* leaf extracts.

In the model of HCA, the data of thirty-seven phenolic compounds in four extractions (Table 1) through standardization were used to conduct analysis, and a hierarchically clustered heat map was obtained as the output (Figure 3C). On the one hand, four extracts were clearly divided into two groups, and the difference between free phenolics and bound phenolics was more significant than that of cultivars, which is consistent with the result of PCA. On the other hand, thirty-seven phenolic compounds were also classified into three groups. For group 1, they were abundant in all samples. Phenolic compounds, including *p*-hydroxybenzoic acid, caffeic acid, *p*-hydroxy-cinnamic acid, and *p*-coumaric acid, from group 2 were only found in bound phenolics, and phenolics of group 3 were almost present in free form. The result of PCA and HCA demonstrated that the bound fraction was indispensable for the evaluation and utilization of phenolic resources in *R. roxburghii* leaves.

### Correlation of Bioactivity With Different Extracts

Table 3 summarizes the correlations between phenolic compounds and bioactivities of leaf extracts from GNN5 and SC. The results showed that the antioxidant activities (DPPH, ABTS, and ORAC) were correlated significantly (*p* < 0.01) with total phenolics (Folin-Ciocalteu) (*r* = 0.958–1.000). Positive high linear correlations (*p* < 0.01) were also observed between the antioxidant activities and total phenolic compounds by UPLC-MS (*r* = 0.972–0.999). IC_50_ was used to express α-glucosidase inhibition, and a smaller IC_50_ value corresponds to stronger α-glucosidase inhibition. Consequently, the negative relationships between IC_50_ value and total phenolics (Folin-Ciocalteu) (*r* = −0.988 to −0.998), and total phenolic compounds (UPLC-MS) (*r* = −0.982 to −0.995) were also observed. The correlations between these bioactivities and phenolic compounds content in *R. roxburghii* leaf extracts were in line with the previous studies (14). The significant and positive correlations between the total phenolic compounds (UPLC-MS) and total phenolics (Folin-Ciocalteu) from SC and GNN5 implied that the method used to extract the phenolic compounds was effective to remove the non-phenolic interfering substances that could react with Folin-Ciocalteu reagent (38). Moreover, the correlation analysis was conducted to evaluate the major phenolic compound contributors to the bioactivities of *R. roxburghii* leaves. The major phenolics of SC and GNN5, quininic acid, brevifolin carboxylic acid, ellagic acid, hyperoside, and isoquercitrin correlated significantly (*p* < 0.01) with the bioactivities (DPPH, ABTS, ORAC, and α-glucosidase inhibition activity) (*r*_*DPPH*_ = 0.948–1.000, *r*_*ABTS*_ = 0.941–0.996, *r*_*ORAC*_ = 0.937–1.000, *r*_*IC*50_ = −0.935 to −0.997). The results revealed that *R. roxburghii* leaves can be sources of natural phenolics for utilization in the development of functional foods and nutraceuticals.

**TABLE 3:** Correlation between phenolic compounds and bioactivities.

Parameters	GNN5	SC
Total phenolics (Folin-Ciocalteu) vs. DPPH	0.992**	1.000**
Total phenolics (Folin-Ciocalteu) vs. ABTS	0.958**	0.998**
Total phenolics (Folin-Ciocalteu) vs. ORAC	0.991**	1.000**
Total phenolics (Folin-Ciocalteu) vs. α-glucosidase inhibition	−0.988**	−0.998**
Total phenolics (Folin-Ciocalteu) vs. Total phenolic compounds (HPLC-MS)	0.966**	0.999**
Total phenolic compounds (HPLC-MS) vs. DPPH	0.989**	0.999**
Total phenolic compounds (HPLC-MS) vs. ABTS	0.972**	0.999**
Total phenolic compounds (HPLC-MS) vs. ORAC	0.991**	0.999**
Total phenolic compounds (HPLC-MS) vs. α-glucosidase inhibition	−0.982**	−0.995**
Ellagic acid vs. DPPH	0.996**	0.999**
Isoquercitrin vs. DPPH	0.999**	0.999**
Hyperoside vs. DPPH	0.948**	0.948**
Quercitrin vs. DPPH	1.000**	1.000**
Brevifolin carboxylic acid vs. DPPH	0.978**	0.997**
Ellagic acid vs. ABTS	0.987**	0.995**
Isoquercitrin vs. ABTS	0.970**	0.995**
Hyperoside vs. ABTS	0.988**	0.965**
Quercitrin vs. ABTS	0.978**	0.996**
Brevifolin carboxylic acid vs. ABTS	0.941**	0.993**
Ellagic acid vs. ORAC	0.994**	0.999**
Isoquercitrin vs. ORAC	0.998**	0.999**
Hyperoside vs. ORAC	0.937**	0.943**
Quercitrin vs. ORAC	0.998**	1.000**
Brevifolin carboxylic acid vs. ORAC	0.981**	0.998**
Ellagic acid vs. IC_50_	−0.989**	−0.997**
Isoquercitrin vs. IC_50_	−0.995**	−0.997**
Hyperoside vs. IC_50_	−0.935**	−0.937**
Quercitrin vs. IC_50_	−0.995**	−0.997**
Brevifolin carboxylic acid vs. IC_50_	−0.975**	−0.997**

Positive correlation (+), negative correlation (−), significant differences: **p < 0.01.

## Conclusion

For the first time, phenolics fractioned into free and bound forms from *R. roxburghii* leaves of two cultivars were studied and their antioxidant and α-glucosidase inhibition activities were evaluated. The profiles of free and bound phenolics and their bioactivities were quite contrasting. The content of free and bound phenolics in GNN5 was 72.71 and 17.75 mg GAE/g DW and in SC was 94.28 and 11.19 mg GAE/g DW, respectively. Flavonoids comprised the maximum phenolics from the free fraction, whereas phenolic acids made a similar contribution for free and bound phenolics. A total of 37 phenolic compounds were identified and quantified by UPLC-Q-Exactive Orbitrap/MS in which ellagic acid, quercitrin, isoquercitrin, and quininic acid were abundant in the free phenolic fraction, while the bound phenolic fraction contained more gallic acid, ellagic acid, and hyperoside. In addition, multivariate analysis revealed that a strong correlation existed between the TPC/individual phenolic compounds and bioactivities of the *R. roxburghii* extracts. Therefore, the characterization and quantification of phenolic compounds in *R. roxburghii* leaves provide a scientific basis for the traditional practices of the leaves in treating different ailments. The data presented in the phenolic profile of *R. roxburghii* leaves clearly reveal that this leaf provides good sources of natural antioxidants for the development of functional foods, nutraceuticals, cosmetics, medicine, or high value-added products.

## Data Availability Statement

The original contributions presented in this study are included in the article/Supplementary Material, further inquiries can be directed to the corresponding author.

## Author Contributions

YY: formal analysis, investigation, data curation, and writing – original draft. WX: visualization. WL: conceptualization and supervision. WH: resources and funding acquisition. RY: writing – review and editing and project administration. All authors contributed to the article and approved the submitted version.

## Conflict of Interest

The authors declare that the research was conducted in the absence of any commercial or financial relationships that could be construed as a potential conflict of interest.

## Publisher’s Note

All claims expressed in this article are solely those of the authors and do not necessarily represent those of their affiliated organizations, or those of the publisher, the editors and the reviewers. Any product that may be evaluated in this article, or claim that may be made by its manufacturer, is not guaranteed or endorsed by the publisher.
