# Supplementary material for: Free and Bound Phenolic Profiles of Rosa roxburghii Tratt Leaves and Their Antioxidant and Inhibitory Effects on α-Glucosidase
Source: Front Nutr. 2022 Jun 28;9:922496. doi: 10.3389/fnut.2022.922496 (PMC9274239; doi:10.3389/fnut.2022.922496)
Supplement: Supplementary file 1 [file Data_Sheet_1.docx]

**Supplementary material**

**Table S1. Identification of phenolic compositions using UPLC–Q–Exactive Orbitrap/MS method**

| **Peak NO.** | **RT**  **(min)** | **Phenolics** | **Formula** | **Ion peak (*m/z*)** | **Fragment** |
| --- | --- | --- | --- | --- | --- |
| 1 | 1.67 | Quininic acid^ab^ | C_7_H_12_O_6_ | 191.0561 | 127.0400, 155.0349 |
| 2 | 3.05 | Gallic acid^ab^ | C_7_H_6_O_5_ | 169.0131 | 125.0233 |
| 3 | 4.53 | (-)-Gallocatechin^ab^ | C_15_H_14_O_7_ | 305.0667 | 125.0232, 179.0344, 219.0660 |
| 4 | 5.05 | Protocatechuic acid^ab^ | C_7_H_6_O_4_ | 153.0181 | 109.0282 |
| 5 | 5.19 | Neochlorogenic acid^ab^ | C_16_H_18_O_9_ | 353.0873 | 191.0549, 235.9254 |
| 6 | 6.20 | Chlorogenic acid^ab^ | C_16_H_18_O_9_ | 353.0875 | 191.0552 |
| 7 | 6.27 | Brevifolin carboxylic acid^a^ | C₁₃H₈O₈ | 291.0148 | 247.0246, 248.0278 |
| 8 | 6.33 | Catechin^ab^ | C_15_H_14_O_6_ | 289.0717 | 245.0813, 205.0495, 125.0229 |
| 9 | 6.43 | Cryptochlorogenic acid^ab^ | C_16_H_18_O_9_ | 353.0878 | 173.0445, 191.0552 |
| 10 | 6.54 | *p*-Hydroxybenzoic acid^ab^ | C_7_H_6_O_3_ | 137.0231 | 109.0277, 93.0345, 65.0396 |
| 11 | 6.76 | Geranium^a^ | C_12_H_20_O_2_ | 951.0756 | 933.0653, 169.0134, 300.9991 |
| 12 | 6.82 | Corilagin^a^ | C_27_H_22_O_18_ | 633.0742 | 275.0198, 300.9991 |
| 13 | 6.89 | 6,7-Dihydroxycoumarin^ab^ | C_9_H_6_O_4_ | 177.0182 | 133.0284, 89.03824 |
| 14 | 7.01 | Caffeic acid^ab^ | C_9_H_8_O_4_ | 179.0337 | 135.0437 |
| 15 | 8.09 | Benzoic acid^ab^ | C_7_H_6_O_2_ | 121.0282 | 77.0380, 94.0281 |
| 16 | 8.20 | Castalagin^a^ | C_41_H_26_O_26_ | 933.0651 | 915.0599, 631.0576, 450.9948 |
| 17 | 8.87 | Vanillin^ab^ | C_8_H_8_O_3_ | 151.0388 | 136.0155 |
| 18 | 8.93 | Rutin^ab^ | C_27_H_30_O_16_ | 609.1461 | 300.0279, 301.0353 |
| 19 | 9.00 | *p*-Hydroxy-cinnamic acid^ab^ | C_9_H_8_O_3_ | 163.0388 | 93.0345, 119.0458 |
| 20 | 9.01 | *p*-Coumaric acid^ab^ | C_9_H_6_O_2_ | 163.0385 | 119.0400 |
| 21 | 9.21 | Ellagic acid^ab^ | C_14_H_6_O_8_ | 300.9990 | 257.0083, 229.0132, 189.7720 |
| 22 | 9.32 | Hyperoside^ab^ | C_21_H_20_O_12_ | 463.0885 | 300.0275, 271.0248 |
| 23 | 9.44 | Isoquercitrin^ab^ | C_21_H_20_O_12_ | 463.0885 | 300.0275 |
| 24 | 9.76 | Ferulic acid^ab^ | C_10_H_10_O_4_ | 193.0497 | 178.0264, 134.0362, 149.0598 |
| 25 | 9.92 | Kaempferol -3-o-rutinoside^ab^ | C_27_H_30_O_15_ | 593.1513 | 285.0404 |
| 26 | 10.03 | Quercetin 3-O-6''-acetylglucoside^a^ | C_23_H_22_O_13_ | 505.0993 | 271.0234, 301.0348 |
| 27 | 10.10 | Proanthocyanidins^ab^ | C_30_H_26_O_13_ | 593.1514 | 285.0404 |
| 28 | 10.28 | Isoferulic acid^ab^ | C_10_H_10_O_4_ | 193.0498 | 178.0264, 134.0362 |
| 29 | 10.55 | Kaemperol-3-O-glucuronide^a^ | C_21_H_18_O_12_ | 461.0717 | 285.0407 |
| 30 | 10.61 | Astragaline^ab^ | C_21_H_20_O_11_ | 447.0931 | 284.0327 |
| 31 | 10.68 | Quercitrin^ab^ | C_21_H_20_O_11_ | 447.0927 | 301.0346 |
| 32 | 11.03 | Kaempferol pentoside^a^ | C_20_H_18_O_10_ | 417.0831 | 285.0389 |
| 33 | 12.28 | Phlorizin^ab^ | C_21_H_24_O_10_ | 435.1296 | 273.0771, 167.0340 |
| 34 | 14.67 | Quercetin^ab^ | C_15_H_10_O_7_ | 301.0353 | 151.0030, 178.9983 |
| 35 | 16.22 | Naringenin^ab^ | C_15_H_12_O_5_ | 271.0608 | 151.0022, 119.0489, 177.0183 |
| 36 | 16.43 | Kaempferol^ab^ | C_15_H_10_O_6_ | 285.0411 | 151.0013 |
| 37 | 16.78 | Isorhamnetin^ab^ | C_16_H_12_O_7_ | 315.0511 | 300.0280 |

^a^ compared with references and OTCML database, ^b^ compared with reference standards. *Product ions were generated by the fragmentation of [M-H]^-^

**Table S2. Calibration curves used for UPLC-MS/MS quantification of polyphenols.**

| Phenolic compounds | Calibration curves | Correlation coefficients (r2) | Linear ranges (ng/mL) |
| --- | --- | --- | --- |
| Quininic acid | Y = 2465290+87257.3*X | 0.9995 | 23.708-4984.517 |
| Gallic acid | Y = 244149+49255.7*X | 0.9998 | 17.626-4990.380 |
| (-)-Gallocatechin | Y = -454748+25740.7*X | 0.9999 | 38.681-4992.880 |
| Protocatechuic acid | Y = 1959580+79473.3*X | 0.9997 | 30.473-4987.805 |
| Neochlorogenic acid | Y = -119075+17857.7*X | 0.9999 | 34.116-4993.843 |
| Chlorogenic acid | Y = 62366.9+32176.3*X | 0.9999 | 34.639-4994.298 |
| Catechin | Y = 1515200+48745.8*X | 0.9997 | 23.529-4994.121 |
| Cryptochlorogenic acid | Y = 474713+33987.7*X | 0.9998 | 26.186-4989.541 |
| p-Hydroxybenzoic acid | Y = 3688590+54438.8*X | 0.9984 | 203.683-4971.669 |
| 6,7-Dihydroxycoumarin | Y = 6647080+177233*X | 0.9990 | 20.360-4975.603 |
| Caffeic acid | Y = 18894500+168117*X | 0.9965 | 216.557-4958.016 |
| Benzoic acid | Y = 535685+8114.79*X | 0.9988 | 4.938-4975.114 |
| Vanillin | Y = -1678920+23679.8*X | 0.9996 | 88.044-5012.153 |
| Rutin | Y = -308640+21668.8*X | 1.0000 | 17.329-5002.319 |
| p-Hydroxy-cinnamic acid | Y = 7740030+120355*X | 0.9986 | 1.160-4973.325 |
| *p*-Coumaric acid | Y = 10876200+111677*X | 0.9941 | 216.313-4940.500 |
| Ellagic acid | Y = 2433540+28802.6*X | 0.9985 | 22.603-4987.252 |
| Hyperoside | Y = 234160+34777*X | 0.9999 | 40.724-4993.357 |
| Isoquercitrin | Y = 1179990+28955.4*X | 0.9988 | 21.387-4973.138 |
| Ferulic acid | Y = 924222+46287*X | 0.9998 | 31.517-4990.871 |
| Kaempferol -3-o-rutinoside | Y = 1096780+23226.6*X | 0.9979 | 9.686-4964.202 |
| Proanthocyanidins | Y = 1101720+23225.4*X | 0.9979 | 9.474-4964.243 |
| Isoferulic acid | Y = -463468+3578.82*X | 1.0000 | 203.975-5000.795 |
| Astragaline | Y = 4480010+55731.8*X | 0.9958 | 214.147-4949.544 |
| Quercitrin | Y = 2724010+37912.9*X | 0.9974 | 178.921-4963.372 |
| Phlorizin | Y = 1674270+32231.2*X | 0.9978 | 8.575-4963.852 |
| Quercetin | Y = 9025650+97962.7*X | 0.9965 | 177.955-4917.970 |
| Naringenin | Y = -158894+644.172*X | 1.0000 | 497.881-4999.735 |
| Kaempferol | Y = 11428900+138226*X | 0.9970 | 228.113-4978.839 |
| Isorhamnetin | Y = 4881440+151993*X | 0.9997 | 43.784-4997.512 |


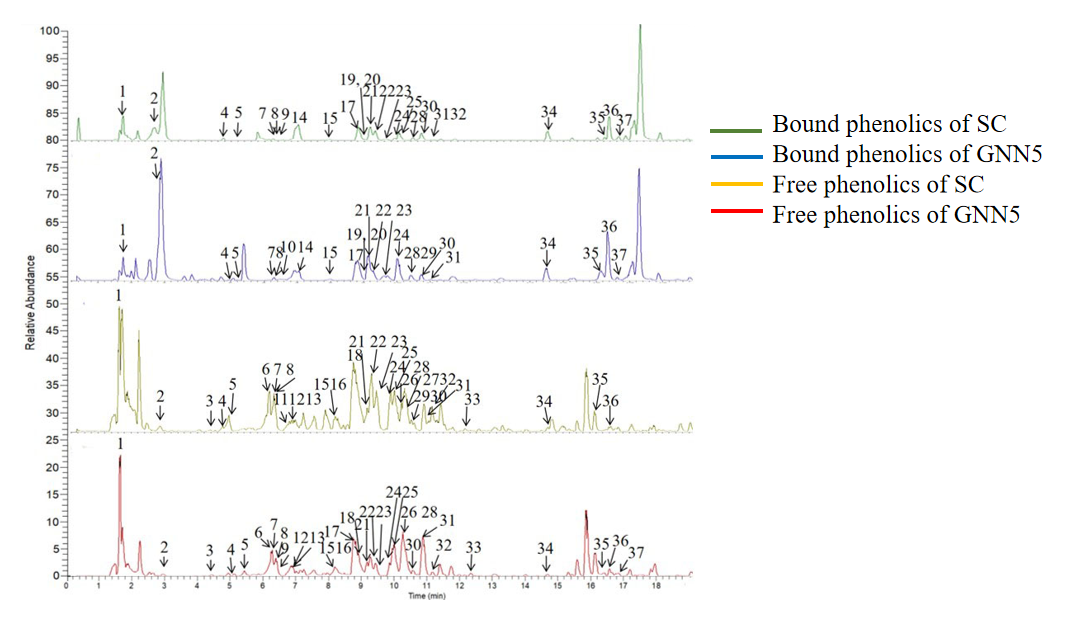
**Fig S1.Total ion chromatograms (TICs) of polyphenols extracts by UPLC-Q-Exactive Orbitrap/MS analysis. Numbers of the identified compounds are those reported in Table 1.**
